# Temporal Trends in Public Interest in Glaucoma: An Infodemiological Analysis Using Google Trends

**DOI:** 10.7759/cureus.116133

**Published:** 2026-09-12

**Authors:** Yuto Yoshida, Masaki Tanito

**Affiliations:** 1 Ophthalmology, Juntendo University School of Medicine, Tokyo, JPN; 2 Ophthalmology, Faculty of Medicine, Shimane University, Izumo, JPN

**Keywords:** glaucoma, glaucoma awareness campaigns, google trends, light up in green, world glaucoma week

## Abstract

Background

In recent years, glaucoma awareness campaigns have been increasingly implemented worldwide. However, the impact of glaucoma awareness campaigns on public interest remains unclear. This study evaluated temporal trends in public interest in glaucoma in Japan and worldwide using Google Trends and explored their potential association with glaucoma awareness campaigns.

Methodology

This infodemiological study used Google Trends to evaluate the temporal changes in public interest in glaucoma. Relative search volume (RSV) for “glaucoma” was obtained in Japan and worldwide from January 2004 to December 2025 and used as a surrogate measure of public interest. Temporal trends in RSV were evaluated using segmented regression analysis with both prespecified breakpoints corresponding to the launch of glaucoma awareness campaigns and data-driven breakpoints estimated from the observed trends.

Results

Temporal trends in RSV were evaluated in Japan and worldwide. Worldwide, the RSV slope significantly increased after the launch of World Glaucoma Week in 2008 (+8.44 points/year, *P*<0.001), with a data-driven breakpoint at 2008. In Japan, the slope significantly increased after the launch of the Light Up in Green campaign in 2015 (+2.24 points/year, *P*<0.001), with a data-driven breakpoint at 2017.

Conclusions

Our results demonstrate an increase in public interest in glaucoma over the past decade in Japan and worldwide, alongside the expansion of glaucoma awareness activities.

## Introduction

Glaucoma is a leading cause of irreversible visual impairment [1,2]. Early detection of glaucoma is essential to prevent disease progression [3]. Therefore, public awareness initiatives that enhance knowledge and understanding of glaucoma and encourage regular eye examinations are important for facilitating its early detection.

World Glaucoma Week is an international initiative to raise awareness of glaucoma that has been observed worldwide since 2008 [4]. Held annually for one week in March, it promotes a variety of awareness activities tailored to local circumstances in participating countries and regions. These activities include free eye examinations, educational lectures, media campaigns, public awareness events, and advocacy efforts directed toward governmental organizations. World Glaucoma Week is promoted internationally by organizations including the World Glaucoma Association.

In Japan, glaucoma awareness activities during World Glaucoma Week have been conducted in parallel with global initiatives. As a unique national initiative, the Light Up in Green campaign was launched in 2015 [5]. The campaign is organized by the Japan Glaucoma Society with the support of the Japan Ophthalmologists Association and the Japanese Ophthalmological Society. From 2015 to 2017, illumination events were primarily held at public facilities across Japan. The campaign was subsequently expanded to medical institutions in 2018 and to other types of facilities in 2020. The number of participating facilities has increased substantially over time, and the initiative has also expanded to selected locations outside Japan [5].

Although these awareness initiatives have been continuously implemented, it remains unclear how public interest in glaucoma has changed in response to these activities. To address this knowledge gap, this study aimed primarily to characterize temporal changes in glaucoma-related Google search interest worldwide and in Japan and to assess whether changes in these trends coincided with the introduction of major glaucoma awareness campaigns; secondary objectives were to compare glaucoma search interest with that for other major ophthalmic diseases and to evaluate seasonal variation in Japan.

## Materials and methods

Study design

This infodemiological study used Google Trends (Google LLC; Mountain View, CA, USA) [6] to evaluate temporal changes in public interest in glaucoma. Google Trends provides aggregated information on the relative frequency of Google searches over time and has been increasingly applied to investigate the population-level information-seeking behavior across various health-related topics [7-12].

Google Trends data were retrieved on August 15, 2026. The study period was set from January 2004 to December 2025, covering the period for which Google Trends data were available. Searches were conducted using the “All categories” setting, and all queries were entered as “search terms.” Two geographic settings were examined: “Worldwide” for the global analysis and “Japan” for the Japan-specific analysis. For the worldwide analysis, the search term “glaucoma” was used, whereas “緑内障” was used for the analysis in Japan. Relative search volume (RSV) for glaucoma was obtained for each region. Google Trends normalizes search interest on a scale from 0 to 100, with a value of 100 representing the highest relative search interest within the specified location and time period. For comparisons between glaucoma and other major ophthalmic diseases, the respective search terms were entered simultaneously within the same Google Trends query to allow comparison under the same normalization framework.

This study used publicly available, anonymized data obtained from Google Trends; therefore, institutional review board approval was not required.

Statistical analysis

Temporal trends in RSV were assessed using segmented linear regression analysis. For the worldwide analysis, 2008, corresponding to the launch of World Glaucoma Week, was specified as a breakpoint, dividing the study period into two segments. For the analysis in Japan, 2008 and 2015, corresponding to the launch of World Glaucoma Week and the Light Up in Green campaign, respectively, were specified as breakpoints, dividing the study period into three segments. Annual slopes and their statistical significance were estimated for each segment. Changes in slope at each breakpoint were also tested to determine whether the temporal trend differed significantly between consecutive segments. In addition, an exploratory segmented regression analysis was performed in which the breakpoint was not prespecified but was estimated directly from the data. This analysis was conducted separately for the worldwide and Japanese glaucoma RSV time series to identify the time point at which the temporal trend changed. To compare search interest in glaucoma with other major eye diseases, RSV trends for diabetic retinopathy, age-related macular degeneration, and retinitis pigmentosa were also examined in Japan and worldwide.

To assess seasonal patterns in search interest in Japan, monthly RSV distributions were examined separately for the periods before (2004-2014) and after (2015-2025) the launch of the Light Up in Green campaign. Within each period, RSV values were grouped by calendar month, and differences in RSV distributions across the 12 months were assessed using the Kruskal-Wallis test. All statistical tests were two-sided, and a P-value<0.05 was considered statistically significant. All analyses were performed using the R software (version 4.5.1; R Foundation for Statistical Computing, Vienna, Austria).

## Results

Figure 1 and Table 1 show the temporal trends in RSV for glaucoma in Japan and worldwide from 2004 to 2025. Worldwide, RSV significantly decreased from 2004 to 2007 (-6.91 RSV/year; P<0.001), followed by a significant increase from 2008 to 2025 (+1.53 RSV/year; P<0.001), with a significant change in slope in 2008 (+8.44 RSV/year; P<0.001). In Japan, no significant trend was observed from 2004 to 2007 (+0.03 RSV/year; P=0.936) or from 2008 to 2014 (+0.76 RSV/year; P=0.062), and the change in slope in 2008 was not significant (+0.73 RSV/year; P=0.143). In contrast, RSV significantly increased from 2015 to 2025 (+3.00 RSV/year; P<0.001), with a significant change in the trend compared with the preceding period (+2.24 RSV/year; P<0.001).

**Figure 1:**
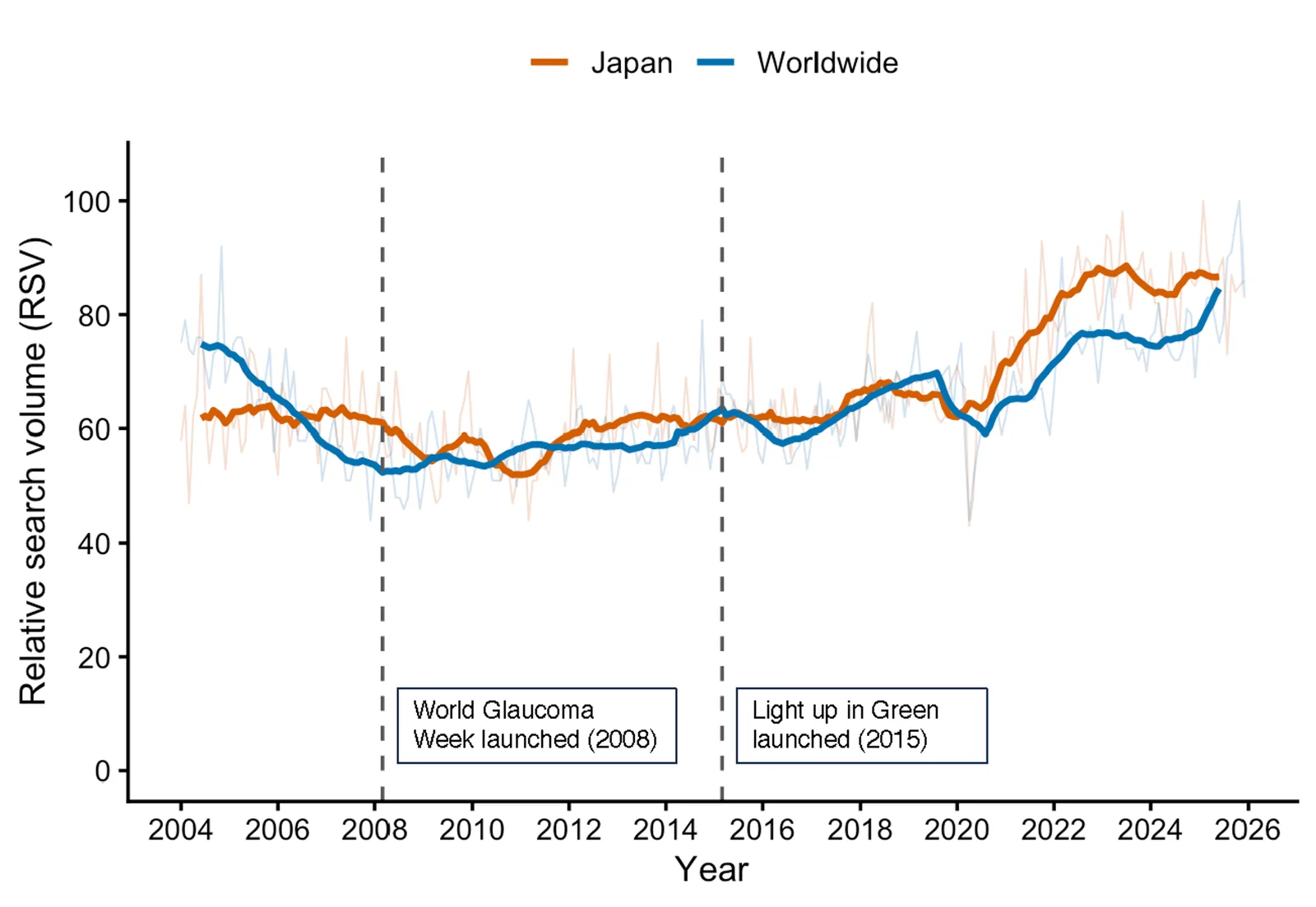
Temporal trends in relative search volume for glaucoma in Japan and worldwide

**Table 1:** Temporal trends and changes in relative search volume for glaucoma in Japan and worldwide Annual trends in RSV and changes in the slope of the trends across the specified periods were evaluated using segmented linear regression analysis. The statistical significance of both the trends and slope changes was determined based on the t-statistic for each regression coefficient, with P-values reported alongside their corresponding test statistics. RSV: relative search volume

Region/period	Annual trend in RSV, point	P-value for trend	Change in slope	P-value for slope change
Worldwide				
2004-2007	-6.91	<0.001	-	-
2008-2025	+1.53	<0.001	+8.44/year	<0.001
Japan				
2004-2007	+0.03	0.936	-	-
2008-2014	+0.76	0.062	+0.73/year	0.143
2015-2025	+3	<0.001	+2.24/year	<0.001

Search interest in glaucoma was consistently higher than that in the other major ophthalmic diseases examined, with a marked increase observed in recent years (Figure 2). Exploratory segmented regression identified a breakpoint in worldwide glaucoma search interest at 2008.46 (95% confidence interval (CI), 2008.00-2008.92). In Japan, a breakpoint was identified at 2017.59 (95% CI, 2016.70-2018.48), after which the upward trend became more pronounced (Figure 2).

**Figure 2:**
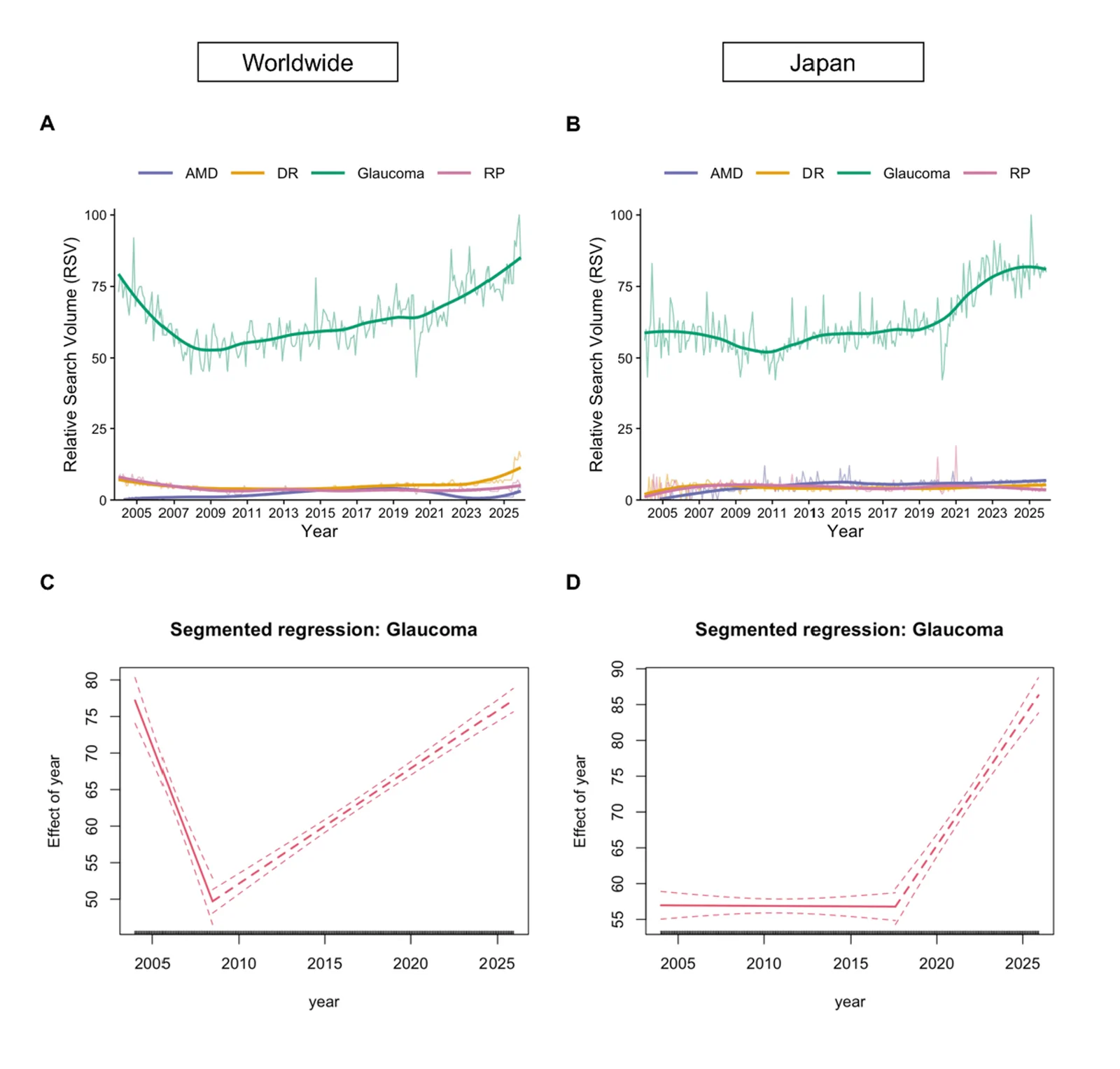
Temporal trends in public interest in glaucoma and other ocular diseases in Japan and worldwide (A) Trends in relative search volume (RSV) for age-related macular degeneration (AMD), diabetic retinopathy (DR), glaucoma, and retinitis pigmentosa (RP) worldwide. (B) Trends in RSV for the same ophthalmic diseases in Japan. (C) Segmented regression analysis of worldwide search interest in glaucoma. The estimated breakpoint was 2008.46 (95% CI, 2008.00-2008.92). (D) Segmented regression analysis of search interest in glaucoma in Japan. The estimated breakpoint was 2017.59 (95% CI, 2016.70-2018.48). The breakpoints indicate the estimated time points at which the slope of RSV changed. CI: confidence interval

Figure 3 shows the monthly distribution of RSV for glaucoma before and after Light Up in Green in Japan. No significant differences in RSV distribution across calendar months were observed either before 2015 (P=0.067) or from 2015 onward (P=0.791).

**Figure 3:**
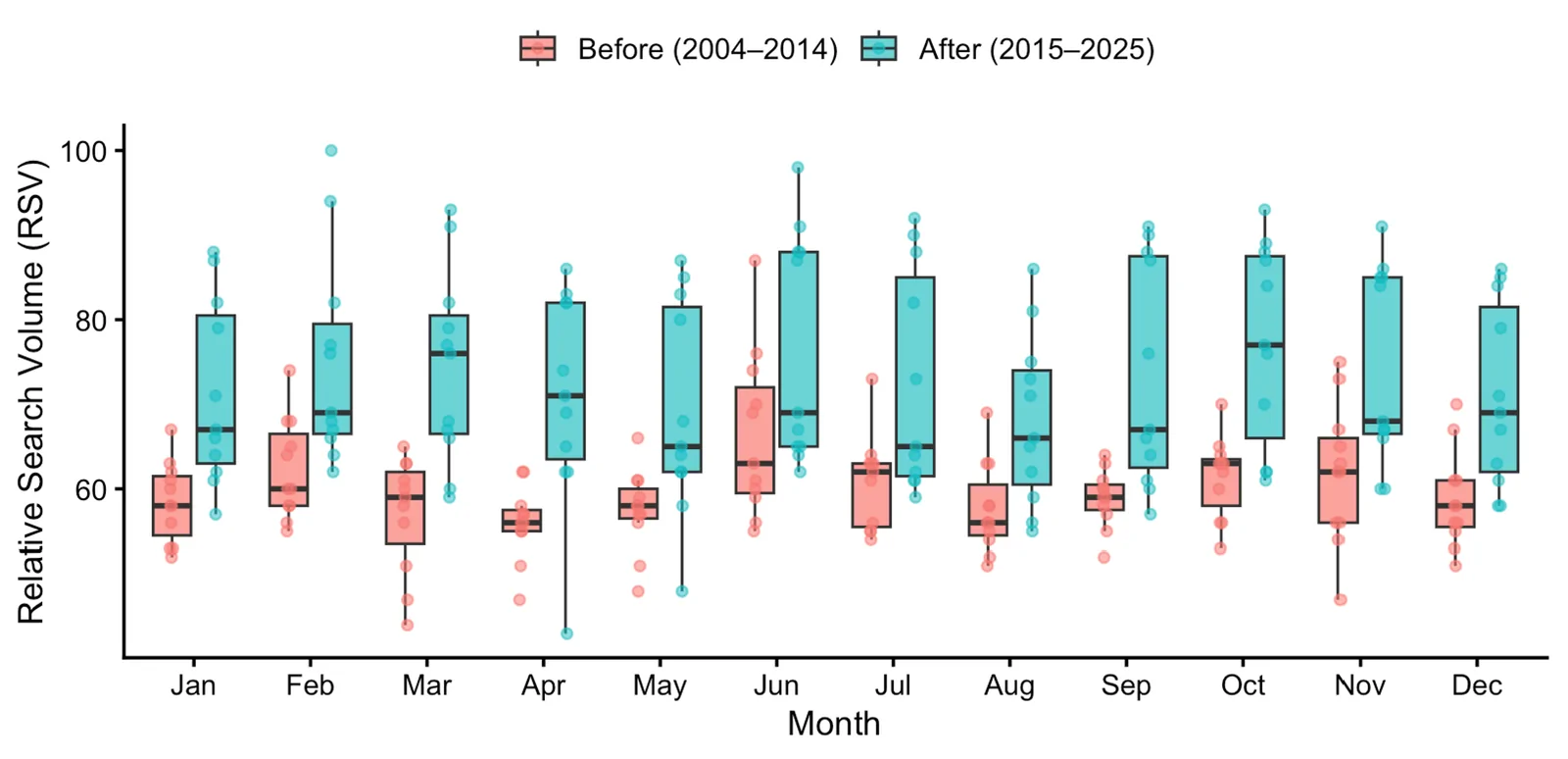
Monthly distribution of relative search volume for glaucoma before and after the Light Up in Green campaign in Japan

## Discussion

This study evaluated temporal trends in public interest in glaucoma in Japan and worldwide. In this study, significant increases in public interest in glaucoma were observed after the introduction of glaucoma awareness campaigns in Japan and worldwide.

The observed increase in public interest may coincide with the expansion of glaucoma awareness activities. Notably, the data-driven breakpoint worldwide closely corresponded to the launch of World Glaucoma Week in 2008, while the breakpoint in Japan occurred a few years after the launch of the Light Up in Green campaign in 2015 (Figure 2). During World Glaucoma Week, a wide range of awareness initiatives are conducted annually worldwide, including public lectures, glaucoma screening programs, and educational activities through various media platforms, such as radio, television, and newspapers [4]. In Japan, the Light Up in Green campaign was launched in 2015 to raise public awareness of the importance of early detection of glaucoma by illuminating buildings and landmarks in green [5]. As shown in Table 2, the number of participating sites increased substantially from only five in 2015 to more than 1,600 in 2025. Similar initiatives have also been undertaken internationally, with prominent landmarks around the world having been illuminated in green, including the Empire State Building in New York, the Sydney Opera House in Australia, and the London Eye in the United Kingdom. The diversification and continued expansion of these awareness activities may have increased opportunities for the public to encounter information about glaucoma, thereby contributing to the observed increase in public interest. However, the causal relationship between glaucoma awareness activities and increased public interest remains unclear, and further studies are needed to clarify this relationship.

**Table 2:** Annual number of facilities participating in the Light Up in Green campaign Permission to use the data was obtained from the Japan Glaucoma Society [5].

Facility type	2015	2016	2017	2018	2019	2020	2021	2022	2023	2024	2025
Public facilities	5	19	37	54	69	55	75	85	90	126	151
Clinics/hospitals	-	1	7	31	81	130	291	425	612	629	640
Other facilities	-	-	-	-	-	45	101	225	485	736	800
Overseas	-	-	-	-	-	-	1	2	7	10	15
Total	5	20	44	85	150	230	468	737	1,194	1,501	1,606

As shown in Figure 2, RSV for glaucoma remained higher than that for the other ophthalmic diseases examined throughout the study period, both worldwide and in Japan. This high level of public interest may partly reflect population aging, given that the prevalence of glaucoma increases with age [13,14]. As populations age, a growing number of individuals may become affected by or concerned about glaucoma, potentially increasing opportunities to seek glaucoma-related information. 

Given that major glaucoma awareness initiatives, including World Glaucoma Week and the Light Up in Green campaign, are primarily conducted in March [4,5], we examined whether RSV showed significant monthly variation. However, no significant differences in monthly RSV distributions were observed even after the initiation of these awareness activities. This finding does not necessarily indicate that the awareness campaigns were ineffective. Glaucoma-related awareness and information dissemination are not limited to March but are conducted throughout the year, which may partly explain the absence of a clear seasonal pattern in search interest. A previous study of other diseases has reported increased search interest during specific awareness months, whereas others have found no clear increase [15], suggesting that the relationship between awareness campaigns and search behavior may vary depending on the disease and the type of awareness activity. Future studies using multiple measures of public interest are needed to identify which types of awareness activities are most effective.

This study has several limitations. First, Google Trends data may be subject to selection bias, as they are limited to internet users, particularly those who use Google to search for information. As internet penetration varies across countries and regions, these differences may have influenced the Google Trends data. Second, changes in RSV may have been influenced by glaucoma-related news, media coverage, and social media activity; therefore, a causal relationship between awareness campaigns and increased search interest cannot be established. Social media is used to disseminate information during World Glaucoma Week, and platforms such as X and Instagram are also utilized as part of the Light Up in Green campaign in Japan. Future studies should quantify awareness activities and examine their associations with RSV to clarify their impact on public interest. Third, RSV is an indirect measure of public interest. Future studies using surveys are needed to directly assess public awareness.

## Conclusions

In conclusion, the diversification and continued expansion of these awareness activities occurred alongside the observed increase in glaucoma-related search interest. Further studies are warranted to elucidate the impact of glaucoma awareness initiatives on public interest in glaucoma.

## References

[1] Jonas JB, Aung T, Bourne RR, Bron AM, Ritch R, Panda-Jonas S (2017). Glaucoma. Lancet.

[2] Tanito M (2025). Proposal of the Glaucoma Etiology Complex (GEC): a structured framework for understanding the multifactorial nature of glaucoma. Cureus.

[3] Weinreb RN, Khaw PT (2004). Primary open-angle glaucoma. Lancet.

[4] (2026). World Glaucoma Association: World Glaucoma Week. http://www.worldglaucomaweek.org/.

[5] (2026). Japan Glaucoma Society: Light Up in Green campaign. https://www.ryokunaisho.jp/light_up/.

[6] (2026). Google Trends. https://trends.google.com/trends/?utm_source=chatgpt.com.

[7] Motosko C, Zakhem G, Ho R, Saadeh P, Hazen A (2018). Using Google to trend patient interest in botulinum toxin and hyaluronic acid fillers. J Drugs Dermatol.

[8] Havelka EM, Mallen CD, Shepherd TA (2020). Using Google Trends to assess the impact of global public health days on online health information seeking behaviour in Central and South America. J Glob Health.

[9] Patel JC, Khurana P, Sharma YK, Kumar B, Sugadev R (2020). Google Trend analysis of climatic zone based Indian severe seasonal sensitive population. BMC Public Health.

[10] Sharma M, Sharma S (2020). The rising number of COVID-19 cases reflecting growing search trend and concern of people: a Google Trend analysis of eight major countries. J Med Syst.

[11] Zitting KM, Lammers-van der Holst HM, Yuan RK, Wang W, Quan SF, Duffy JF (2021). Google Trends reveals increases in internet searches for insomnia during the 2019 coronavirus disease (COVID-19) global pandemic. J Clin Sleep Med.

[12] Keitoku K, Nishimura Y, Hagiya H, Koyama T, Otsuka F (2021). Impact of the World Antimicrobial Awareness Week on public interest between 2015 and 2020: a Google Trends analysis. Int J Infect Dis.

[13] Tham YC, Li X, Wong TY, Quigley HA, Aung T, Cheng CY (2014). Global prevalence of glaucoma and projections of glaucoma burden through 2040: a systematic review and meta-analysis. Ophthalmology.

[14] Iwase A, Suzuki Y, Araie M (2004). The prevalence of primary open-angle glaucoma in Japanese: the Tajimi Study. Ophthalmology.

[15] Nishimura Y, Acoba JD (2022). Impact of breast cancer awareness month on public interest in the United States between 2012 and 2021: a Google Trends analysis. Cancers (Basel).

